# Photochemically induced ischemic stroke in rats

**DOI:** 10.1186/2040-7378-4-13

**Published:** 2012-08-09

**Authors:** Antje Schmidt, Maike Hoppen, Jan-Kolja Strecker, Kai Diederich, Wolf-Rüdiger Schäbitz, Matthias Schilling, Jens Minnerup

**Affiliations:** 1 Department of Neurology, University of Münster, Albert-Schweitzer-Campus 1, Gebäude A1, 48149, Münster, Germany; 2 EVK Bielefeld, Bethel, Neurologische Klinik, Burgsteig 13, 33617, Bielefeld, Germany

**Keywords:** Photothrombosis, Experimental stroke, Animal model

## Abstract

**Background:**

Photothrombosis was introduced as a model of ischemic stroke by Watson et al. in 1985. In the present paper, we describe a protocol to induce photothrombotic infarcts in rats.

**Findings:**

The photosensitive dye Bengal Rose is intravenously administered and a laser beam is stereotactically positioned onto the skull. Illumination through the intact skull leads to local activation of Bengal Rose, which results in free radical formation, disturbance of endothelial function and thrombus formation in illuminated small cortical vessels.

**Conclusions:**

Photochemically induced infarcts cause long-term sensorimotor deficits, allow long-term survival and are particularly suitable to assess the effectiveness of neuroregenerative therapies in chronic stroke studies.

## Background

Stroke is the second most frequent cause of death and a leading cause of disability and cognitive impairment in developed countries [1,2]. Ischemic stroke, which accounts for approximately 80% of strokes, constitutes a huge socioeconomic burden. Experimental models of cerebral ischemia have been developed to mimic human stroke, gain a better understanding of underlying pathophysiological mechanisms and foster the development of new therapies. Common models of focal and multifocal cerebral ischemia include the intraluminal thread middle cerebral artery occlusion (MCAo) model, surgical MCAo models using ligation, clipping, electrocauterization etc., endothelin-1-induced MCAo, and embolization models using blood clots or other embolus material for vessel occlusion (for review see [3]).

In 1985, Watson et al. introduced photothrombosis as a technique to induce focal cerebral infarction in the cortical vasculature of rats [4]. The key mechanism is a photochemical reaction triggered by systemic administration of Bengal Rose and focal illumination of the skull. Illumination leads to local activation of Bengal Rose, which results in free radical formation, disturbance of endothelial function and local thrombosis in small cortical vessels [3,5]. As compared to other animal models of stroke, animal preparation is simple because it does not require mechanical manipulation of cerebral blood vessels or parenchyma. Lesion size and location can be modulated by altering the irradiating intensity, duration of light exposure, beam position, and dye concentration. Apart from that, photothrombotic infarcts do not impede long-term survival and are thus suitable for chronic stroke studies [4,6].

## Materials and methods

### Materials

Animals

Adult male Wistar rats (250 g)

Reagents

S-ketamine hydrochloride 25 mg/ml (CEVA, Düsseldorf, D)

2% Xylazine (CEVA, Düsseldorf, D)

Bengal Rose (Sigma Aldrich, Steinheim, D)

4% paraformaldehyde (Merck KGaA, Darmstadt, D)

Equipment

Stereotactic device (TSE Systems, Bad Homburg, D)

Laser EAGLE 60 series (G Laser Technologies, Kleinostheim, D)

Polyethylene catheter 30 m, 0,58 mm inner diameter (Smith Medical, Grasbrunn, D)

Heating device TKM-0902 (FMI, Backnang, D)

Surgical instruments: Scissors, Forceps, Razor, Stitching Set (Bayha, Tuttlingen, D)

### Methods

All animal experiments must comply with national and institutional animal welfare regulations and require approval of an appropriate ethics committee. Our experimental protocols were appoved by our local ethics committee.

**Figure 1:**
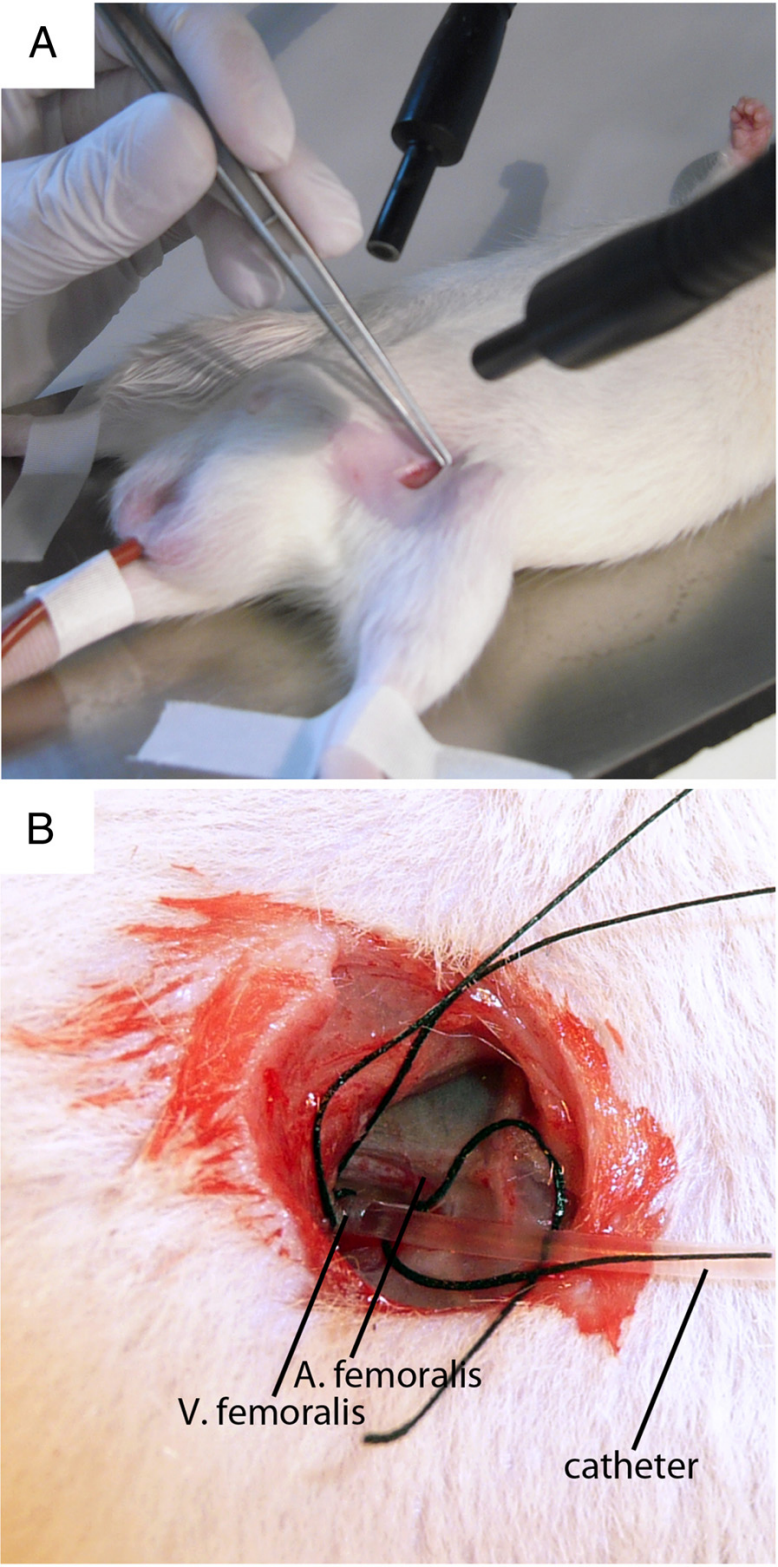
**A: The left femoral vein is exposed.B**: A polyethylene catheter is inserted and fixed with threads.

**Figure 2:**
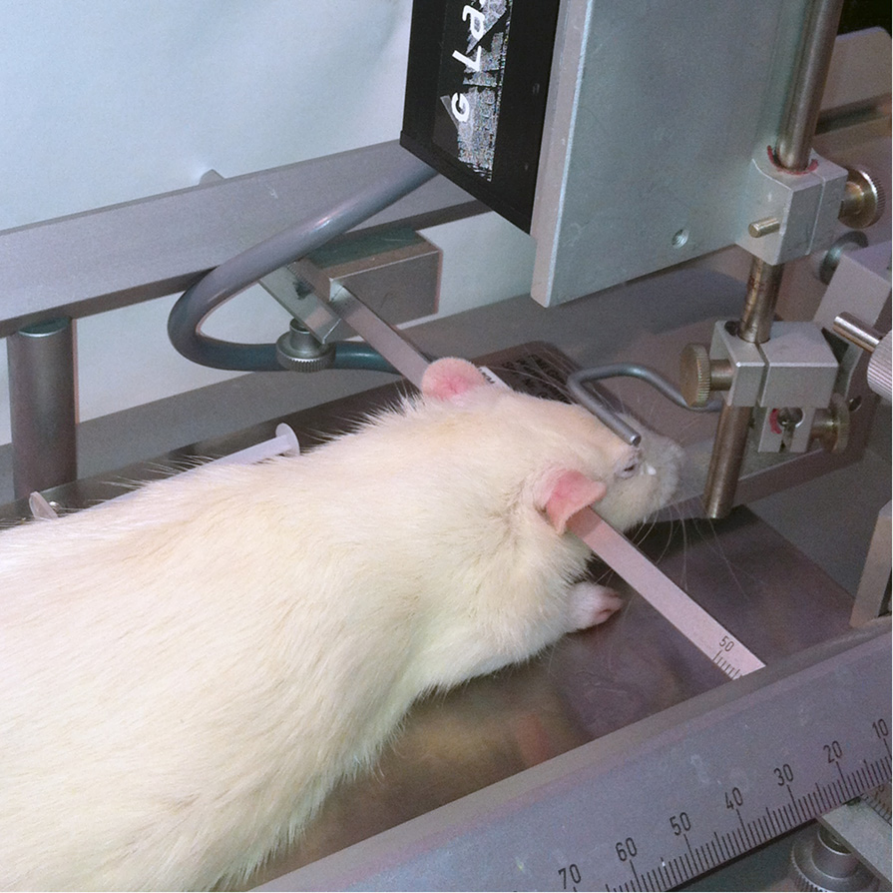
A rat in a stereotactic frame.

**Figure 3:**
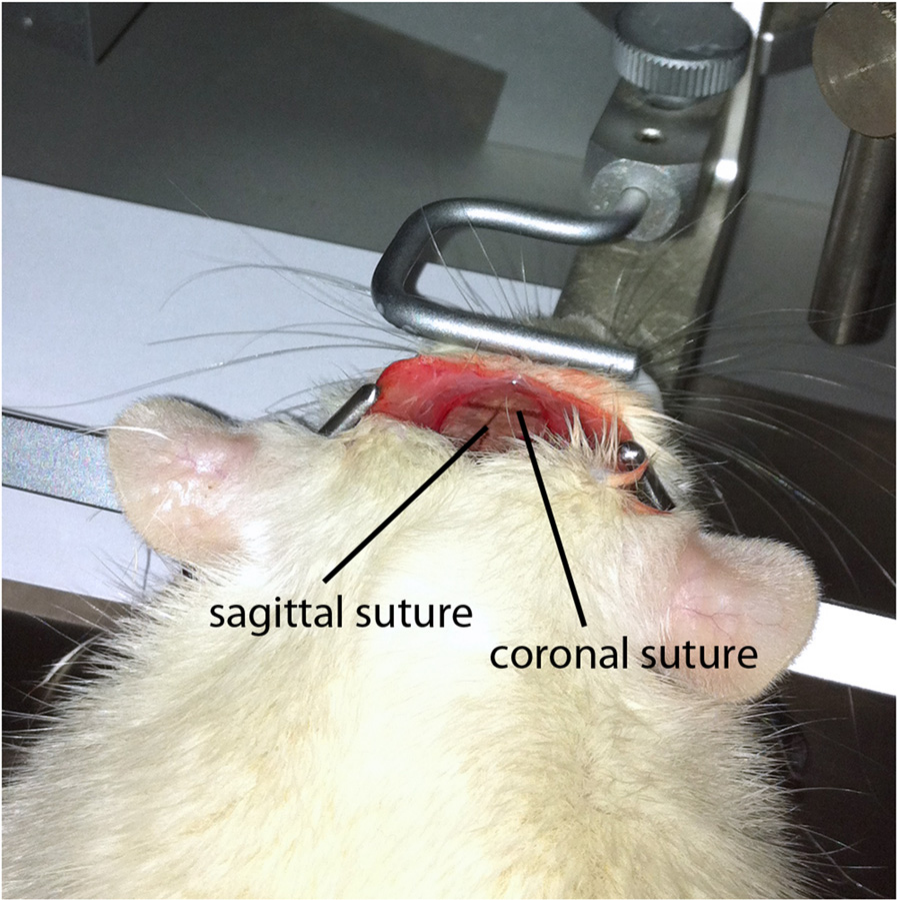
**Skull exposure is achieved by a longitudinal scalp incision.** Coronal and sagittal sutures are easily identified.

**Figure 4:**
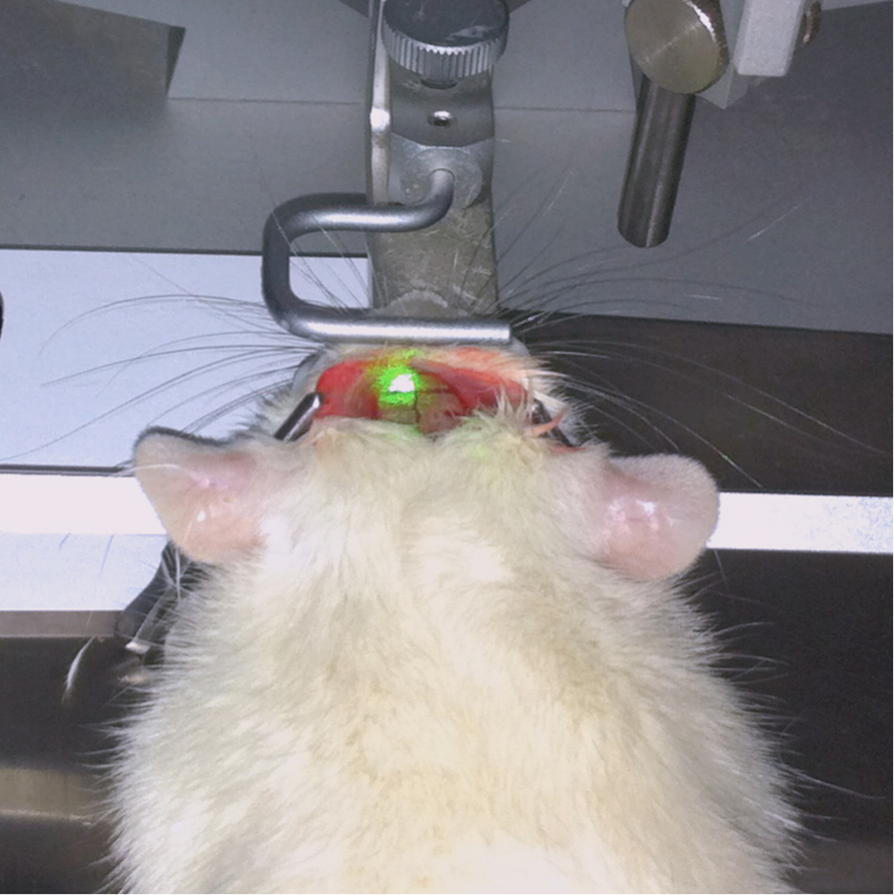
**A laser beam is stereotactically positioned onto the skull and the skull is illuminated for 20 minutes.** During the first 2 minutes of illumination, Bengal rose is intravenously administered.

1. The rats are anesthetized with an intraperitoneal injection of ketamine hydrochloride (100 mg/kg body weight; Ketanest) and xylazine hydrochloride (8 mg/kg body weight). The depth of anesthesia is verified by foot pinches. If the rats are adequately anesthetized, there is no response of extremity (flexion or withdrawal).

2. The body temperature is monitored and maintained at 37°C by a thermostatically controlled heating pad.

3. The left femoral vein is exposed and cannulated with a polyethylene catheter for Bengal Rose infusion. The catheter is fixed with threads (Figure 1).

4. The rats are placed in a stereotactic frame (Figure 2).

5. The scalp is longitudinally incised (2.0-2.5 cm) and retracted to expose the skull. To avoid wound complications the skull exposure should be achieved with a single cut.

6. The periost is gently removed and coronal and sagittal sutures are identified (Figure 3).

7. A laser beam of 8 mm diameter (G Laser Technologies) and 560 nm wavelength is stereotactically positioned onto the skull 0.5 mm anterior to the bregma and 3.5 mm lateral from the midline (Figure 4).

8. The skull is illuminated for 20 minutes. During the first 2 minutes of illumination, Bengal Rose (0.133 mL/kg body weight, 10 mg/mL saline) is slowly injected through the previously inserted catheter.

9. Afterwards, the catheter is withdrawn and the skin is sutured.

10. After the surgical procedures, the rats are returned to their cages in a temperature-controlled room with 12-h light and dark cycles and food and water ad libitum.

11. Sham-operated animals receive the same treatment including Bengal Rose injection, but without illumination of the skull.

Timing

Step 1: 8 - 12 min.

Step 2: 1 min.

Step 3: 8 - 12 min.

Step 4: 2 - 4 min.

Step 5: 1 - 2 min.

Step 6: 1 - 2 min.

Step 7: 1 min.

Step 8: 20 min.

Step 9: 8 - 12 min.

### Outcome

**Figure 5:**
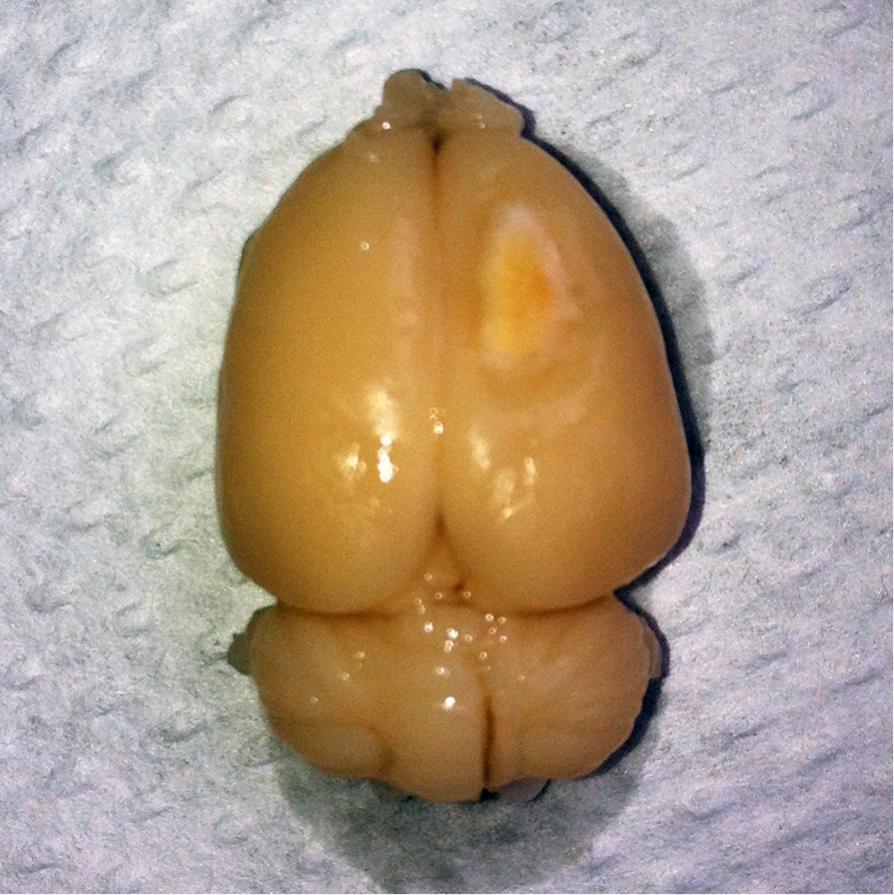
Rat brain 28 days after photochemically induced ischemic stroke.

**Figure 6:**
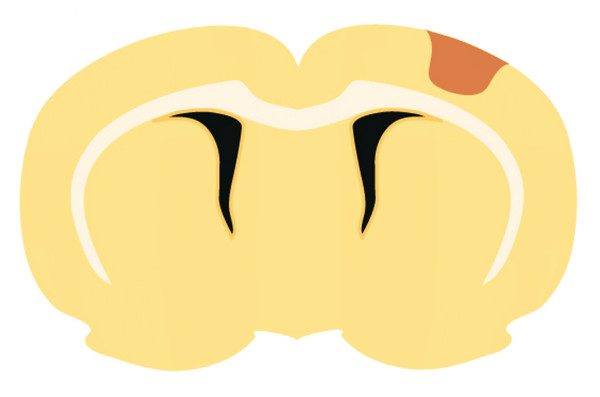
A schematic of a photochemically induced cortical infarct.

The major outcome measures in preclinical stroke studies are infarct size and neurological deficits. Infarct volumes are determined either histologically from stained brain sections or non-invasively with MRI scans. In photochemically induced stroke, which is characterized by a rapidly evolving ischemic damage without salvageable penumbra, the infarct size is relatively stable and hardly responsive to neuroprotective therapies [7]. Figures 5 and 6 illustrate size and location of photochemically induced infarcts. The severity of sensorimotor deficits can be assessed by a battery of behavioural tests, e.g. the cylinder test, the adhesive tape removal test, the forelimb placing test, the beam balance test, etc. (for review see [8]). Photothrombotic infarcts cause mild to moderate, long-term sensorimotor deficits, particularly well displayed by the cylinder test and the adhesive tape removal test [9,10]. In our model, sensorimotor deficits are evident for at least four weeks [9,10]. In addition, neurological scoring systems have been established to provide an overall assessment of the animal´s condition. However, the most commonly used neurological scoring systems are not sensitive enough to reliably reflect sensorimotor deficits subsequent to photochemically induced stroke. The Morris water maze is applied to assess cognitive deficits [11]. Multiple histological analyses can be performed in order to elucidate molecular and cellular changes, including immunohistochemical stainings to analyze neurogenesis, dendritic and axonal plasticity, the postischemic inflammatory reaction, the upregulation of neurotransmitters, etc.

Advantages

Animal preparation is simple and requires neither craniotomy nor mechanical manipulation of cerebral blood vessels or parenchyma [4].

Lesion size and location can be modulated by altering the irradiating intensity, duration of light exposure, beam position, and dye concentration [4].

 Lesion size and location are highly reproducible [5, 6].

The procedure-associated mortality rate is low (< 10%).

Animals exhibit long-term sensorimotor deficits [9, 10].

Photothrombotic infarcts allow long-term survival [4].

Disadvantages

Photothrombotic infarcts severely affect vessel walls and cause an early vasogenic edema atypical of human stroke [6].

The penumbra is missing in this model [3].

Due to the cortical location and the relatively small lesion size, sensorimotor deficits are minor in comparison to other stroke models [6].

## Conclusions

Photothrombotic infarcts cause long-term sensorimotor deficits and allow long-term survival in chronic stroke studies. The infarct size is hardly responsive to neuroprotective therapies. However, the lack of responsiveness to neuroprotection in the acute phase allows a differentiation between neuroprotective and neuroregenerative effects, and, as a consequence, neuroregenerative therapies can be evaluated more precisely. Altogether, photochemically induced infarcts are particularly suitable to analyse the effectiveness of neuroregenerative therapies.

## Competing interests

The authors declare that they have no competing interests.

## Authors’ contributions

AS and MH carried out the experiments. AS drafted the manuscript. JM and MS supervised the experiments and helped drafting the manuscript. MH, JKS, KD and WRS contributed to conception and design and revised the manuscript. All authors read and approved the final manuscript.

## Authors’ information

JM and MS shared senior authorship.

## References

[B1] Donnan GA Fisher M Macleod M Davis SM Stroke Lancet 2008 371 1612 1623 10.1016/S0140-6736(08)60694-7 18468545

[B2] Lopez AD Mathers CD Ezzati M Jamison DT Murray CJ Global and regional burden of disease and risk factors, 2001: systematic analysis of population health data Lancet 2006 367 1747 1757 10.1016/S0140-6736(06)68770-9 16731270

[B3] Braeuninger S Kleinschnitz C Rodent models of focal cerebral ischemia: procedural pitfalls and translational problems Exp Transl Stroke Med 2009 1 8 10.1186/2040-7378-1-8 20150986 PMC2820446

[B4] Watson BD Dietrich WD Busto R Wachtel MS Ginsberg MD Induction of reproducible brain infarction by photochemically initiated thrombosis Ann Neurol 1985 17 497 504 10.1002/ana.410170513 4004172

[B5] Schroeter M Jander S Stoll G Non-invasive induction of focal cerebral ischemia in mice by photothrombosis of cortical microvessels: characterization of inflammatory responses J Neurosci Methods 2002 117 43 49 10.1016/S0165-0270(02)00072-9 12084563

[B6] Macrae IM Preclinical stroke research–advantages and disadvantages of the most common rodent models of focal ischaemia Br J Pharmacol 2011 164 1062 1078 10.1111/j.1476-5381.2011.01398.x 21457227 PMC3229752

[B7] Diederich K Quennet V Bauer H Muller HD Wersching H Schabitz WR Minnerup J Sommer C Successful Regeneration After Experimental Stroke by Granulocyte-Colony Stimulating Factor Is Not Further Enhanced by Constraint-Induced Movement Therapy Either in Concurrent or in Sequential Combination Therapy Stroke 2012 43 185 192 10.1161/STROKEAHA.111.622159 22020031

[B8] Schallert T Behavioral tests for preclinical intervention assessment NeuroRx 2006 3 497 504 10.1016/j.nurx.2006.08.001 17012064 PMC3593401

[B9] Minnerup J Kim JB Schmidt A Diederich K Bauer H Schilling M Strecker JK Ringelstein EB Sommer C Scholer HR Schabitz WR Effects of neural progenitor cells on sensorimotor recovery and endogenous repair mechanisms after photothrombotic stroke Stroke 2011 42 1757 1763 10.1161/STROKEAHA.110.599282 21566228

[B10] Minnerup J Seeger FH Kuhnert K Diederich K Schilling M Dimmeler S Schabitz WR Intracarotid administration of human bone marrow mononuclear cells in rat photothrombotic ischemia Exp Transl Stroke Med 2010 2 3 10.1186/2040-7378-2-3 20298535 PMC2828442

[B11] Morris R Developments of a water-maze procedure for studying spatial learning in the rat J Neurosci Methods 1984 11 47 60 10.1016/0165-0270(84)90007-4 6471907

